# Enhancing the Integration of Pre-Emptive Pharmacogenetic (PGx) Testing in Primary Care: Prioritizing Underserved Patients’ Preferences in Implementation

**DOI:** 10.3390/jpm14121128

**Published:** 2024-11-29

**Authors:** Emma G. Bryan, Kelsey Lunsford, Michaela D. Mullis, Antionette McFarlane, Erica Elwood, Brian E. Gawronski, Julio D. Duarte, Carla L. Fisher

**Affiliations:** 1 Department of Health Outcomes and Biomedical Informatics, College of Medicine, University of Florida, Gainesville, FL 32610, USA; emma.bryan@ufl.edu (E.G.B.); mullis.md@ufl.edu (M.D.M.); 2 Department of Advertising, College of Journalism and Communications, University of Florida, Gainesville, FL 32511, USA; kelseylunsford@ufl.edu; 3 Department of Public Health Sciences, School of Medicine, University of Virginia, Charlottesville, VA 22903, USA; rkv4fp@virginia.edu; 4 Department of Pharmacotherapy and Translational Research, College of Pharmacy, University of Florida, Gainesville, FL 32610, USA; erica.elwood@cop.ufl.edu (E.E.); briangaw@ufl.edu (B.E.G.); 5 Center for Pharmacogenomics and Precision Medicine, University of Florida, Gainesville, FL 32610, USA

**Keywords:** pharmacogenetic testing, patient preferences, implementation, primary care

## Abstract

**Background/Objectives**: The integration of pharmacogenetic (PGx) testing into primary care has not been widely implemented, despite its benefits for patients and providers. PGx testing could also reduce health disparities as patients with lower healthcare access are prescribed higher proportions of medications with PGx guidelines. Little is known about the preferences of patients who have experienced PGx testing to inform implementation across the care process. This qualitative study aimed to refine implementation by capturing patient preferences on (1) testing and prescription timing, (2) patient–clinician discussion of results during post-test counseling, and (3) usability of a card during results dissemination. **Methods**: Interviews were conducted with 25 primary care patients from clinics primarily serving medically underserved populations. Interview transcripts were thematically analyzed using a constant comparative approach. **Results**: While patients supported both reactive and pre-emptive testing, they valued pre-emptive PGx testing because it is *proactive for future health needs*, *expedites treatment*, and is *convenient*. Patients’ preferences for receiving prescriptions depended on several factors: *having immediate access to needed medications, avoiding experiencing medication side effects and interactions*, *avoiding taking ineffective medications*, and *avoiding inconveniences*. Patients identified three issues critical to patient–clinician interactions when receiving testing results: *information specific to medications*, *clarification and further information about their results*, and *enhanced clinician accessibility related to the results*. Lastly, they liked that the results card could *facilitate discussions with clinicians and was informative* and *convenient* but said it lacked *clarity*. **Conclusions**: These findings should inform implementation strategies for integrating PGx testing in primary care for underserved patients.

## 1. Introduction

Pharmacogenetic (PGx) testing is garnering global recognition as a personalized care component that can enhance health outcomes by ensuring patients are prescribed medications that are more effective in treating their condition(s) and have a lower risk of adverse effects. PGx testing may also reduce health disparities and promote equitable care as patients with lower access to healthcare are prescribed a higher proportion of medications with PGx guidelines available, and this especially is true for racially minoritized patients, who also encounter more side effects [1,2]. Although the benefits of PGx testing for both patients and providers have been established, implementation of this personalized care component has been slow moving into practice, especially in the primary care setting [3]. Implementing PGx testing into primary care may be particularly beneficial as these clinicians routinely prescribe medications with PGx guidelines available, and would allow their patients to receive more personalized care.

While various models exist for clinically implementing PGx testing [4,5,6,7], less is known about patients’ implementation preferences for PGx testing in the primary care setting. A recent scoping review of what does exist showed that much of this research focuses on pre-test counseling, where clinicians must address individual factors shaping patients’ uptake of PGx testing, like their motivations for completing the testing and the perceived benefits [4]. This evidence has contributed to pre-test counseling guidelines for clinicians to use when discussing with patients the personal benefits, risks, and limitations of PGx testing, as well as explaining what PGx testing is and how it works [7,8,9].

Yet little attention has been paid to the perspectives of patients who have received PGx testing and their preferences for implementation across the care process. Understanding these perspectives is crucial for developing best practices in primary care settings [9]. For instance, less is known about patients’ timing preferences for testing initiation and receiving prescriptions, and studies indicate no consensus on patients’ preferences for pre-emptive (i.e., routine) testing versus diagnostic (i.e., reactive) testing [10]. Additionally, while implementation models highlight the importance of communicating PGx testing results to primary care physicians [11], the patients themselves also stress the importance of talking to a clinician to understand the results, a component that can be left to the discretion of the provider [5,8,12]. Although post-test counseling is essential to patients’ understanding of their results and informed decision-making processes, a scoping review noted that it has not received sufficient attention in the implementation process [4]. Furthermore, how patients receive their results (e.g., in person, via electronic chart) has implications for how they might then use those results. For instance, studies have indicated that PGx testing location can restrict patients’ and clinicians’ accessibility to the results, warranting the development of new approaches to results dissemination [11,12,13].

It is imperative to understand patients’ preferences regarding PGx testing implementation in the primary care setting and to promote equity in care by prioritizing the voices of underserved, minoritized, and marginalized patient populations. Such research can inform the development of best practices for implementation across the testing process while also recognizing that such practices should be targeted based on the clinical populations each healthcare practice serves. With these overarching goals in mind, we explored patients’ preferences to refine future PGx testing implementation by focusing on three areas of implementation: (1) testing and prescription timing, (2) patient–clinician discussion of results during the post-test counseling phase, and (3) usability of a results card during results dissemination.

## 2. Materials and Methods

### 2.1. Participant Recruitment

**Figure 1:**
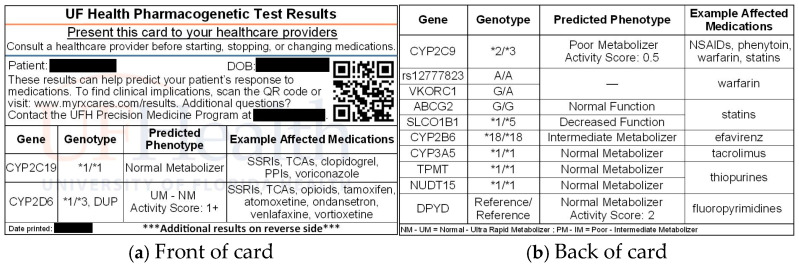
Card patients received with PGx test results.

This qualitative study used an interview method with participants who were recruited from a clinical trial (Trial of Preemptive Pharmacogenetics in Underserved Patients (TOPP-UP), NCT05141019) evaluating the implementation of PGx testing of medically underserved patients in primary care within a large healthcare system in Florida. Participants were recruited from three clinics primarily serving medically underserved patient populations and randomized to receive either pre-emptive pharmacogenetic testing with a multi-gene panel (listed in Figure 1) or standard care, which involved no testing during the trial period. Participants who received pre-emptive PGx testing received their results both within their electronic health record and on a wallet-sized (3 7/16 in × 2 1/8 in) printed and laminated results card (Figure 1). Inclusion criteria for the trial included being an adult with (1) a medication prescribed for a condition potentially treated by a medication with PGx guidelines available, (2) at least 3 active prescriptions within their medical record, and (3) a documented medication change within the 8 months prior to enrollment. Trial participants who were randomized to the pre-emptive PGx testing arm were asked if they would be willing to participate in a post-trial interview. After receiving approval from the University of Florida’s Institutional Review Board (IRB202101316), participants were invited to schedule an interview and efforts were made to have comparable representation by race and clinic site.

### 2.2. Data Collection

Individual in-depth interviews were conducted and audio-recorded via phone or Zoom by an author trained in qualitative methodology and with expertise in interviewing medically underserved patients [A.M.]. Interviews were conducted using a semi-structured guide, which was developed by an expert in qualitative methodology and implementation science [C.L.F.] and refined by multiple team members with expertise in PGx testing and primary care [E.E., B.G., J.D.D.] (see Appendix A for the interview guide). The interview questions aimed to gather participants’ perspectives on the three components of PGx testing implementation design: testing and prescription timing, patient–provider interaction, and results dissemination. Each interview lasted about 30 min and resulted in 208 pages of transcribed data.

### 2.3. Data Analysis

Data were managed in ATLAS.ti, and a thematic analysis was conducted using a constant comparative method (CCM) approach [14]. To ensure and maintain rigor, an expert in thematic analysis [M.D.M.] led the analysis with additional trained coders [E.B., K.L.], and a lead qualitative expert [C.L.F.] oversaw the entire coding process [15]. The analysis included four main steps informed by the CCM: (1) reading all transcripts for data immersion, (2) open coding to assign labels to patterns emergent for each aim, (3) collapsing codes into categories to identify themes and to establish a typology for each aim, and (4) axial coding to establish thematic properties to further define each theme. A codebook was developed using a modified rapid analysis approach [16,17], led by the lead analyst [M.D.M.] and overseen by C.L.F., with analysis conducted concurrently with data collection to promote thematic saturation and expedite the coding process. Additional interviews were also conducted to confirm the extent of the saturation of themes in the codebook [15]. Additional coders [E.B., K.L.] then validated and further refined the codebook once data collection ended to refine each typology of themes and properties, which was overseen by C.L.F. to ensure trustworthiness. All but one theme was reported by participants from all three clinic sites. On average, themes were reported by at least 43.3% of participants, with saturation extending up to 72%. Verification strategies to promote rigor were employed across the design (purposively sampling, conducting concurrent data collection and analysis to identify the extent of saturation, using multiple coders, and ensuring methodological coherence) [15]. Results (themes and thematic properties) are also presented in translational tables using an ecological sentence synthesis approach rather than a listing of themes and properties [18,19]. This approach puts the findings into action statements that can easily be read to promote the translation of findings into practice. Collectively, the tables provide directed guidance that can inform health professionals’ ability to integrate PGx testing into primary care practice [18,19,20].

## 3. Results

### 3.1. Participants

A total of 25 participants (19 female, 6 male) who had received PGx testing were interviewed. Participants were racially diverse, with 13 identifying as White/European, 11 as Black/African American, and 1 as mixed race. Most participants reported being “very comfortable” (*n* = 9) or “somewhat comfortable” (*n* = 9) with understanding medical terminology and concepts. Participants were, on average, about 60 years old and ranged in age from 38 to 77. The majority were not employed (*n* = 20), with 11 reporting being retired and 9 reporting being disabled. Of the 18 participants who reported income, 56% reported a household income of USD 42,000 or less annually, a relatively low income level. Additionally, 32% reported an education level of high school diploma/GED or less, while 60% had completed either some college (*n* = 8) or an associate degree (*n* = 7). See Table 1 for full demographics.

### 3.2. Preferences for Testing and Prescription Timing

#### 3.2.1. Timing of PGx Testing

Participants were asked if they would like PGx testing to be offered pre-emptively as a part of routine care or only when needed for a specific condition (i.e., reactive or diagnostic testing). While these two approaches were explained by the moderator during the interview, it was noteworthy that participants at times seemed to struggle with fully comprehending the meaning of pre-emptive testing, with some voicing misunderstanding of pre-emptive testing (*n* = 10). However, collectively, nearly half (*n* = 12) voiced support for having PGx testing implemented as part of routine care. Participants described three factors informing their support of timing PGx testing pre-emptively (see Table 2).

First, they appreciated that pre-emptive PGx testing helped them to be *proactive for future health needs*. Specifically, they wanted their doctors to have their PGx testing results available to address and improve treatment of future unknown health issues: “If anything pops up in the future, something else, they will have the tools to prescribe me the best medicine possible” (Black female, age 52).

Participants also viewed pre-emptive testing as an opportunity to *expedite treatment*. Making testing a part of routine care would help them avoid wait times for their PGx testing results. Consequently, they could avoid a delay in being prescribed effective medication:

“In my opinion, to have it in place, to be able to just have [it] available in the chart, because then that would certainly expedite the ability for the person to have the best treatment at the time that the discussion was going on about it rather than wait.” (White female, age 71).

Lastly, participants found pre-emptive PGx testing *convenient*. They liked the idea of PGx testing being completed at the earliest opportunity, rather than needing to return to their doctor’s office to have PGx testing performed when a health issue arose. As one patient expressed, pre-emptive testing could help him avoid a long unwanted drive to the doctor:

“I would prefer that my [primary] doctor do it because he’s only 35–40 min away. Whereas if I go to [city where specialist is], it’s a good two hours. I’ve been doing that for eight months, and I’m so burned out on going [there] I hope I never see it again.” (White male, age 69).

#### 3.2.2. Timing of Prescription

Participants were asked if they would prefer to receive prescribed medications for a condition before or after receiving their PGx testing results. While some participants voiced a misunderstanding of the difference between receiving prescriptions before and receiving prescriptions after PGx testing results (*n* = 6), collectively, participants described support for both options. They identified various factors that informed which option they would prefer (see Table 2).

Only one factor informed why they would want a prescription before receiving their PGx testing results: *wanting immediate access to needed medications*. Specifically, they wanted to avoid a delay in receiving medications while waiting for their PGx testing results, especially if they were experiencing significant adverse health issues that a medication could address:

“I wouldn’t want to wait for six months or something like that for testing to come in in order to do it. If it was just a few days to a couple of weeks and I wasn’t like [in] agonizing pain or something—like a medication that you use for pain relief. Some pain relief is better than no pain relief while you’re waiting.” (White female, age 64).

Three factors informed patients’ support of receiving prescriptions after their PGx testing results were disseminated. First, if participants waited until their results were back, they could *avoid experiencing medication side effects and interactions*. In general, they wanted to ensure they were not prescribed medications that would cause a negative reaction. As a participant explained,

“I reacted to so many different medicines. Even though I can take it, I always react and have a lot of side effects to a lot of the different medicines that they do prescribe sometimes. So, if they could kind of wait and see what the results say, maybe I don’t. … I’m thinking that maybe they can find, get the right, medicine, you know?” (Black female, age 68).

Additionally, participants wanted to *avoid taking ineffective medications* that would not adequately treat their health conditions and, therefore, be unnecessary to take: “With either option, it would be mainly to verify whether or not I am metabolizing a specific medication properly. … Because if it’s not going to be therapeutic for me, I wouldn’t want to be taking it” (White female, age 50).

Lastly, participants noted that they could *avoid inconveniences* by waiting to receive prescribed medications until after receiving their PGx testing results. These inconveniences included having to later switch medications if their results indicated a change was needed, as well as incurring additional costs. As a participant remarked, “I would rather have them wait so that I know. That makes the most sense. Because, I mean, why incur the expense of a very expensive medication and then have to stop taking it?” (White female, age 70).

### 3.3. Preferences for Patient–Clinician Discussion About PGx Testing Results

Participants were asked for feedback on the clinical communication of results. A total of 14 participants reported having talked to their clinician about their results. It is noteworthy that, although 11 reported that they did not have an opportunity to discuss their PGx testing results with their clinician, all of these individuals expressed that they wanted a clinical discussion or appointment to address their results. Participants identified three issues that were critical to the patient–clinician interaction when receiving their PGx testing results. Participants wanted (1) *information specific to medications*, (2) *clarification and further information about their results*, and (3) *enhanced clinician accessibility related to the results* (see Table 3). Participants also further provided specifics within each area as to why the clinical conversation was critical.

#### 3.3.1. Information Specific to Medications

Participants wanted to discuss with a clinician about how their PGx testing results would inform their individual treatment plan (i.e., information about medications). They noted two specific issues they wanted to discuss regarding receiving information about medications.

First, it was important that participants could converse with clinicians to *decide optimal medications and changes in prescriptions*. Specifically, they wanted to know whether their PGx results indicated a need to adjust their medications or confirmed they were on the right medication: “I remember that I was relieved that those medications were adequate for the condition. … because the genetic testing or because of the information that was there, it seemed to be adequately prescribed” (White female, age 71).

Second, participants explained that they wanted their clinician to *provide information related to medication effects*. They appreciated clinicians taking the time to discuss how their PGx test results explained why certain medications were (in)effective, had side effects, or led to drug interactions. A participant who had discussed this with her clinician explained:

“I liked it because … you know most doctors stay in for like 10 to 15 min and then they’re out. But with that, with the pharmaceutical testing, [the clinician] actually sat there over 25, 30, almost 30–35 min and talked and discussed [it], going over the results and the different medications and why my body was acting the way it was.” (Black female, age 52).

Participants described how addressing medication effects with their clinician not only contextualized clinical decisions to adjust their treatment plan, but also validated their personal health experiences and overall health:

“There were several medications on there that I had been taking for years, one particular being an antidepressant … It was one of the drugs that my body didn’t recognize. … And the fact that I had been on that medication for so many years and I couldn’t figure out why my depression wasn’t getting any better <laughs>, it was like it was my fault that I wasn’t getting better! But I’m on a different one now and things are going <laughs> much, much better.” (White female, age 68).

#### 3.3.2. Clarification and Further Information About Results

Participants wanted their clinician to provide further information and clarity to help them understand their testing results. They identified two reasons why a clinical discussion was important in this regard.

First, participants explained how they needed the clinician to *clarify the meaning of results information on the results card*. They wanted clinicians to clarify information on the card itself so that they could understand their own results better, noting the use of clinical or scientific jargon:

“I would probably want a little bit more in detail about what the genotypes are, and what they’re being tested for. I don’t understand! I’m not a doctor so I can’t read the genotype testings results and the categories that well.” (White male, age 48).

Another participant further explained the importance of clarity so they could interpret the results as they were presented on the card:

“Have [the clinician] read the card and explain to us what the results mean because my husband was interested in that, but I can’t read that stuff. That’d be a good idea. Take it to the primary care and have him explain.” (Mixed Race male, age 75).

One way participants expressed that clinicians could clarify the meaning of their results was by *answering their questions about their results*. Participants not only desired answers, but also expected their clinicians to be prepared to respond to any questions adequately:

“I’m not saying it’s a waste of time, but it’s like it’s a waste of time to ask her because she’s supposed to be a physician. … I don’t know these hard words. … I don’t know about prescription names and stuff. … But I feel like it was hard on her to figure out what it was.” (Black female, age 47).

In sum, while having their questions answered to clarify their results was important, participants also emphasized an inter-related issue concerning clinicians’ knowledge and understanding of PGx testing, which complicated the clinicians’ ability to answer patients’ questions.

#### 3.3.3. Enhanced Clinician Accessibility

As was alluded to in the previous finding, participants explained that, in order for clinicians to provide adequate clarification and additional information about their results, clinicians’ accessibility was critical. They explained the importance of clinician accessibility in two ways.

First, participants stressed the importance of accessibility in terms of the clinicians’ own knowledge and understanding of PGx testing. Thus, clinicians need to *have better clinical comprehension* of PGx results. Participants at times explained that their clinicians did not seem to fully understand PGx testing and results, which inhibited their communication about the results and, therefore, their ability to receive quality personalized care:

“I just happened to show [my clinician] the card for them to not prescribe the wrong medication, and she told me that she don’t really know much about these things. But I feel like if you don’t—I mean, you guys are the one who prescribe medication. You’re supposed to know about these things because it says on the card what I’m not supposed to take or I’m allergic or I have a reaction or something.” (Black female, age 47).

Second, participants noted the importance of clinicians having *access to their results* in their electronic patient portal (i.e., electronic patient chart) to facilitate their interaction about their results. They also indicated the importance of this feature in promoting their communication with other clinicians who could utilize the results in their care decisions:

“[The platform] records everything, pretty much, that’s going on across the spectrum of the different physicians that you’re involved with. So, they all have the ability to read the chart. The primary care physician is where I originally was. That clinic was where I originally had the testing done, and that was where I had the discussion with [the] doctor. … Because in the past, you know how one doctor prescribes a medication, but it’s another doctor from another place and they don’t know unless you tell them that you’ve got that medication or that it’s prescribed. So, by having it in the system across the spectrum of the doctors, makes it much more advantageous to the patient.” (White female, age 71).

### 3.4. Perspectives on PGx Testing Results Card Usability

Participants were asked for their perceptions on the potential usability of receiving a printed card that displayed their PGx testing results. Participants were mostly positive about the card, noting three things they liked that pertained to its usability: (1) *the card can help facilitate discussions with clinicians*, (2) *the card is informative*, and (3) *the card is convenient*. Participants also identified a dislike, which they described as inhibiting the card’s usability: (4) *a lack of clarity* (see Table 4).

#### 3.4.1. Card Can Help Facilitate Discussion with Clinicians

Participants shared that the PGx testing results card was particularly useful as it could prompt discussions about medication decisions with their clinicians. Specifically, participants mentioned two ways the card could be used in this way.

First, participants noted that the card could be used to share information *during primary care communication*. They shared how they either planned to take it to their medical appointments (with various clinicians) or had already used it during appointments to make sure the doctor had the information: “As I recall, as I mentioned, that I actually handed [my clinician] the card and he looked at it and read it, and he seemed to be satisfied with what was there. So, I was satisfied” (White female, age 71). They also noted that having a results card may be important if the clinician does not have access to their results within their online system: “I think it’s very useful. I do have it. … if I need to show it to my primary doctor … because I’m not sure if they have that in their computer or not” (Black female, age 69).

Second, they explained how the card could be used *during clinical discussions with specialists*—providers they do not frequently see. A participant explained, saying, “I have to talk to a surgeon now, so they’re going to want to know because they’ll probably want to try opiates on me” (White female, age 57). Participants also emphasized how the card could facilitate conversations with their pharmacists, considering their expertise and close involvement with drug prescriptions. For instance, a participant shared how she used the card in this way:

“When I go actually to pick up my medicine from the pharmacist, I show it to them and I find that the pharmacy, the pharmacists, they’re very helpful [and] glad that I have it. So, when I actually start asking the pharmacist about different side effects and everything, actually the pharmacies are more helpful than the doctors when they see this card and I show it to them.” (Black female, age 52).

#### 3.4.2. Card Is Informative

Participants explained that the PGx testing results card was informative, which enhanced the card’s usability. Notably, participants who expressed liking the informativeness of the card (*n* = 10) had higher education levels within the study sample, with seven of these participants having either an associate or bachelor’s degree. They described two informational components that promoted the card’s usability.

Participants appreciated the detailed nature of the card, which had *all their results information present*. They particularly liked how the card provided details about how their genes are expressed within their body: “The amount of information that’s on it. I like the fact that it’s easy to tell what the phenotype is” (White female, age 50).

Additionally, participants appreciated when the results card had *personalized details* to clarify how the information on the results card pertained to their individual treatment plans. This included seeing the classes of medication impacted by their phenotype:

“On the front it lists SSRIs, TCAs, opioids, tamoxifen, … venlafaxine, which I take, that is an intermediate metabolizer of those medications, so it shows some of the information I just thought was interesting.” (White female, age 59).

Some participants desired further personalization of the card, wanting it to list all their current prescriptions and show how their genotype impacted the efficacy of their medications:

“Maybe if there was a cross-reference of what meds I’m on and then if it were to say, ‘This is not a good medicine for you’ or ‘This might not be as effective’. If somehow my [medication] list was in there.” (White female, age 38).

#### 3.4.3. Card Is Convenient

Regarding usability, participants also found the card to be convenient. They focused on three factors that promoted its convenience.

First, the card was convenient due to its *size*. Participants explained that because the card is small enough to fit inside of a wallet, it was easy to carry with them at all times and prevent it from getting lost:

“The size is good because I was just able to stick it with my credit cards to have it easily there for my doctor. … I would’ve lost track of a piece of paper, so it was nice to have, because I had it on me in my purse with my credit cards.” (White female, age 38).

Second, participants mentioned that the results card was convenient as it was *easily accessible for others to find*, if needed. This ease of access was highlighted as being useful in emergency situations, where someone else may need to find the card:

“Just having that information and being able to give it to somebody, especially in an emergency situation, because I’m sure they go through your wallet, look for your insurance card and things like that. So having that with my insurance card and my driver’s license, it’s part of who I am.” (White female, age 68).

Participants also mentioned that the card was convenient because it could be *easily shared with other clinicians*, thereby promoting clinicians’ convenient access to the information it provided:

“It’s helpful for me because I have a disability and I don’t have to worry about having, you know, carrying paper or a folder with me. I usually just can pull out everything on my phone and just show, you know, the pharmacy or the doctors. It’s easier access, I guess you could say.” (Black female, age 52).

#### 3.4.4. A Lack of Clarity

Participants identified one barrier to the card’s usability, which centered on a lack of clarity in how the results were presented on the card itself. This lack of clarity inhibited participants’ ability to use it. They described this barrier in three ways.

First, they noted *readability* was an issue. This included challenges with the card’s formatting and their ability to easily read the information. As one patient explained, “The print is small, and I have poor vision” (White female, age 50). Using an organized, structured layout to present the results could enhance readability. As one participant noted, “It was set up in grids very well to be understood” (White male, age 48).

Participants also noted *comprehension* challenges with the results card, specifically having difficulty understanding the information presented on it. They disclosed that comprehension was especially challenging given their lack of familiarity with PGx testing. Some shared how comprehension was key to their ability to use the card and discuss results with their clinician:

“When you don’t understand something, you don’t know how to ask nothing. You know, you do and you don’t, especially when it’s something brand new, and it’s something that you never heard [of]. I’ve heard of it, but never saw anything like it, so I wouldn’t know how to take that and make a question to ask nobody. … You just got a bunch of stuff up here about genes and metabolizers and stuff, and I say, well, I don’t know what all of that means! So, I cannot go into anything about trying to find a question to ask anybody about something. I really, I have to sit and learn something about it and be able to ask a question about it, so I don’t know how to do that.” (Black female, age 68).

Participants further explained that medical jargon (e.g., gene names, active ingredient medication names, drug category names) impeded their comprehension of the card, which reduced its usability:

“I don’t understand a lot of the medicine things. I don’t even understand really what the full names are of them. … I remember looking at [the card] and thinking ‘Hmm. I guess my doctor would understand the medical terms on it.” (White female, age 59).

Interestingly, participants offered a suggestion to address this comprehension barrier and clarify the results presented on the card. They recommended including a resource (e.g., a chart or pamphlet) to guide their interpretation:

“Maybe have some type of chart to better understand what the different things mean. Like it has the gene, the different types of gene, and then it has the normal function, decreased function, intermediate. But I’m not sure, I guess on that first one, it had the CYP, some letters and numbers, and I wasn’t sure if that is my gene, and then I’m relating that to just those letters and numbers to the results of the medication. So I don’t really understand how to read it, I guess.” (White female, age 66).

## 4. Discussion

These findings contribute one of the first studies focused on understanding underrepresented patients’ preferences for PGx testing implementation in the primary care setting. Furthermore, these findings characterize the preferences of patients who may face more challenges with PGx comprehension, as our participants reported having comprehension challenges about the timing of testing and prescriptions, as well as having difficulty interpreting results. These findings may translate to similar diverse populations who report similar characteristics, including lower socioeconomic status and lower education levels. These findings also support the importance of patient education across the implementation process—a factor that has been previously identified as critical to the implementation process [10]. Thus, our study furthers the argument for more targeted approaches to PGx implementation to effectively personalize care and meet each patient populations’ needs.

The findings also uncover patients’ preferences for implementation of PGx testing in the primary care setting and, therefore, highlight key strategies for implementation and testing on a larger scale. Regarding the timing of testing and prescriptions, previous research indicated a lack of consensus among patients, particularly about whether they wanted pre-emptive/routine testing in primary care or diagnostic/reactive testing [10]. Our findings indicate support for pre-emptive testing and extend previous research by providing further explanation as to why (e.g., to be proactive, expedite treatment, convenience). While patients indicated differing opinions on prescription timing (before or after results are received), our findings explain this lack of consensus. Specifically, patients’ preferences depend on individual or situational factors. These findings support the notion that primary care providers must personalize their approach to PGx testing implementation by providing patients with a choice on prescription timing in particular.

Additionally, comprehension of results emerged as a critical issue in the post-test counseling phase, further demonstrating the importance of education in PGx testing implementation across the care process for both patients and clinicians. The importance of clinicians’ communication of results to their patients should be underscored in our findings. Patients want their clinicians to communicate with them so they can understand their results; however, patients do not always receive this communication, and their clinicians are not always equipped to navigate these critical discussions.

Our findings suggest that clinician education/training should be integrated as an implementation strategy and include two major components: (1) education about PGx testing and (2) communication skills training. Previous qualitative and quantitative research supports our observations, reporting that primary care clinicians see value in PGx implementation but lack understanding of how to interpret and apply PGx results in clinical practice [21,22,23,24,25]. Their lack of comprehension is understandable, given that pharmacogenetics remains inadequately integrated into current formal science curricula [25]. Preliminary research shows the effectiveness of additional training for healthcare professions in increasing self-efficacy and competencies in interpreting PGx data [26], and implementation scientists and PGx experts have advocated for such education (e.g., courses, seminars, and online modalities) [27,28]. Our findings extend this research as the patients themselves identified clinicians’ lack of understanding, which may inhibit their ability to explain the meaning of their test results. Thus, we would advocate for primary care providers to receive PGx education that helps them understand the PGx results themselves so that they can integrate them into care and explain them to their patients to inform their shared decision making.

Additionally, based on our findings, we would advocate for clinician education/training to include a component about patient–clinician communication that includes communication skills training. This educational component should include education on the critical role clinicians play in disseminating and explaining PGx testing results to their patients with guidance on what to address during those clinical encounters based on findings from our study (Table 3). Additionally, clinicians should receive communication skills training (CST) on how to facilitate these conversations, as doing so is aligned with better patient and clinical outcomes [29]. This communication training component should include applications of skills in the following key areas identified in our study to address implementation more comprehensively: (1) making shared decisions about testing and prescription timing; (2) sharing results in a comprehensible manner (e.g., explaining jargon); (3) facilitating dialogue and questions about their results; and (4) prioritizing personalized care in results dissemination by providing patients with choices that enhance their usability of the results (e.g., receiving them in person, via card, and/or in their electronic chart) [30,31,32]. To promote the feasibility of implementing clinician training as a key implementation strategy (both in terms of reducing cost and recognizing clinicians’ time constraints), asynchronous online delivery of clinician training should be considered so they can complete it at their convenience as well as refer back to it as needed to promote sustainability of PGx testing in their clinical practice.

In addition to primary care providers’ training, education on PGx testing and talking with patients is also critical for community pharmacists. As participants in our study reported, they talked to their pharmacists about their PGx testing results and some described their pharmacists as being more engaged about pharmacogenetic medicine in comparison to their primary care provider. Yet research has recently shown that pharmacists lack and need PGx education, and this is especially true for pharmacists in community pharmacy practices who lack the resources/benefits of clinic-based pharmacists in larger health institutions [28]. This is problematic given that community pharmacists have more regular day-to-day engagement with patients, and primary care providers sometimes recommend that their patients talk to their pharmacists to better understand their results [21,23,24]. Thus, community pharmacists would benefit from the same training we advocate for primary care providers.

Similarly, a results card might serve as a conversation tool or prompt to initiate this clinical communication and guide discussions about PGx results and what they mean for the patient individually. Patients reported valuing the cards for this reason, believing they could prompt dialogue with primary care providers and sharing examples of how the card was used in this manner. Because the card is informative and easy to share, patients also reported that the card would promote their communication with physicians in other healthcare settings beyond primary care, demonstrating the potential impact of the card in promoting multi-disciplinary care (e.g., specialty care, pharmacy). Future studies might investigate the utility of the card as an intervention strategy for clinical communication while also working to refine the card based on patients’ feedback that the information on the card should be presented in a clear, comprehensible manner to promote usability.

### Limitations

While this study provides valuable insights into the implementation of PGx testing into routine primary care, it is important to acknowledge some limitations, specifically concerning our participant sample, that may impact the interpretation of the findings, as well as their generalizability to a broader population given the goal of the qualitative, targeted design for implementation within a specific population. First, our sample consisted of participants who were mostly women. Additionally, most of our participants identified their race as either White or Black, meaning we did not have racial or ethnic (e.g., Hispanic) representation in our results. Next, our participants were primarily later-midlife- to later-life-aged adults. Future research should explore perspectives from individuals of various age ranges. Findings could also be used to conduct a larger study using a survey approach to identify how these preferences extend to a more general population. Finally, our sample represents just one region in Florida with predominantly rural and suburban residents. While the findings may translate to similar populations, additional research should seek urban-dwelling adults.

## 5. Conclusions

This study identified the preferences of underserved patients to inform and facilitate PGx implementation in primary care settings. Importantly, patients’ perspectives help inform best practices for implementation across the testing process. These areas include testing and prescription timing, patient–clinician discussion of results during post-test counseling, and the usability of a card during results dissemination. The findings should inform future implementation of PGx testing in primary care for underserved patient populations.

## Figures and Tables

**Table 1:** Participant demographics.

Characteristic	*N*	Percent	Mean (Range)
Age	25	100%	59.68 (38–77)
38–49	4	16%	
50–59	8	32%	
60–69	9	36%	
70–77	4	16%	
Gender			
Male	6	24%	
Female	19	76%	
Ethnicity			
Hispanic or Latino	0	0%	
Race			
White	13	52%	
Black	11	44%	
Mixed Race	1	4%	
Marital Status			
Divorced, separated, widowed	9	36%	
Married or member of unmarried couple	8	32%	
Single	8	32%	
Current employment status			
Disabled	9	36%	
Retired	11	44%	
Full time	4	16%	
Part time	1	4%	
Highest educational level attained			
Less than high school (9th grade)	1	4%	
Some high school	1	4%	
High school diploma/GED	6	24%	
Some college	8	32%	
Associate degree	7	28%	
Bachelor’s degree	1	4%	
Postgraduate degree	1	4%	
Estimated yearly household income			
<USD 18,000	5	20%	
USD 18,001–26,000	4	16%	
USD 26,001–34,000	2	8%	
USD 34,001–42,000	3	12%	
USD 42,001–71,000	3	12%	
USD 71,001–100,000	1	4%	
Don’t know/Refuse to answer	7	28%	
Comfort understanding medical terminology/concepts			
Extremely comfortable	4	16%	
Very comfortable	9	36%	
Somewhat comfortable	10	40%	
Neutral	1	4%	
Somewhat uncomfortable	1	4%	

**Table 2:** Patients’ preferences for integrating PGx testing into primary care.

**Patients support the integration of**	**because it ensures patients can**
pre-emptive testing	- be proactive for future health needs; - expedite treatment [if the need arises in the future]; - make testing more convenient.
receiving prescriptions before receiving their PGx test results	- have immediate access to needed medications.
receiving prescriptions after receiving their PGx test results	- avoid medication side effects and interactions; - avoid taking ineffective medications; - avoid inconveniences with prescriptions.

**Table 3:** Patients’ preferences for clinical communication of PGx test results.

**During patient**–**clinician discussions about** **PGx test results, patients want clinicians to**	**to attend to patients’ specific concerns about**
discuss information specific to medications	- finding optimal medications and changing prescriptions;
	- having medication effects (e.g., side effects, efficacy).
clarify and provide further information about the results	- the meaning of results (e.g., explaining jargon);
	- questions they have about the results.
have enhanced accessibility to the results	- their clinicians’ ability to understand results;
	- their clinicians’ ability to access to results.

**Table 4:** Patients’ perspectives on PGx testing results card usability.

**Patients perceive that the results card is usable because it**
can facilitate the patient–clinician discussion with primary care providers and specialists; is informative, with all of the results included and personalized details; is convenient because it is small enough to comfortably carry around and easily accessible for others to find; can be easily shared with other clinicians; is clear, with a design/format that enhances readability and information presented in an understandable way.

## Data Availability

The data presented in this study are available on reasonable request from the corresponding author. The data are not publicly available due to participant privacy.

## Appendix A

Patient Interview Guide

1. One of the things we’d like to improve on is how you receive your results. I’d like to ask you some questions about that. You may already know that it can take anywhere from 3–14 days to receive pharmacogenetic test results. It just depends on the lab that runs the test. Because of this, your doctor will typically have to do one of two things. They would either wait to prescribe your medication until the test results are ready or prescribe a medication and then later possibly change your prescription if the test results indicate your medication should be changed.
  a. What are your thoughts on these two scenarios? Did you experience one or the other?
  b. What do you like about each option and what don’t you like?
  c. Assuming the costs are the same, would you prefer one option from your doctor over the other? Why?
2. There are two other scenarios that physicians could do as well that I’d appreciate your insight on. In addition to doing the testing to identify medications for a specific condition when you need it, sometimes physicians will order additional pharmacogenetic testing on your DNA to identify the best medications for other conditions—conditions you don’t have now but may or may not develop in the future—so that they have the information they need should it be needed in the future. There’s also another scenario: a primary care physician could do the pharmacogenetic testing as a pre-emptive option, meaning they do the testing now as a part of routine care, before you have any conditions, so that if and when the time comes, they have the information they need to prescribe you with the best medications for you personally.
  a. Assuming the costs are the same, would you be interested in these options?
3. When you think back to when you received your pharmacogenetic test results, did you discuss the results with your doctor?
  a. Please share with me what you recall about how your doctor gave you your results.
  b. What did you like about that interaction? What didn’t you like?
  c. Was there anything that was missing during that appointment when you receive your results that you would’ve like to have had?
4. Thinking back to this appointment, what questions did you have for your doctor? Did you ask them?
  a. Please share how the doctor responded to you.
  b. Thinking back to the results, were they understandable to you?
  c. What information could we provide you to enhance your understanding of the results?
5. When receiving your pharmacogenetic test results, what information was most important to you that you wanted to receive?
  a. What other information is important to you related to your results?
  b. Are there other uses for this information that is important to you?
6. How useful do you find the PGx result card that you received as part of this study?
  a. What do you like about this card? Dislike?
  b. Have you shared this information with other doctors that you have seen?
